# Measurement and evaluation of surgical models of spinal fusion in mice, rats, and rabbits

**DOI:** 10.1002/ame2.70215

**Published:** 2026-06-01

**Authors:** Jianghong Lu, Yicun Hu, Xin Yao

**Affiliations:** ^1^ Department of Orthopedics Genertec Universal Xi’an Aero‐Engine Hospital Xi’an Shaanxi China; ^2^ Department of Orthopedics Second Hospital of Lanzhou University Lanzhou Gansu China; ^3^ Department of Day Surgery Center, Honghui Hospital Xi'an Jiaotong University Xi'an Shaanxi China

**Keywords:** Lα intervertebral disc, mice, operation, rabbit, rat, spinal fusion surgery model

## Abstract

This study aims to establish a theoretical foundation for selecting and constructing animal models in clinical research on spine‐related diseases. By establishing surgical models in three experimental animal species—mice, rats, and rabbits—we compare their respective advantages and disadvantages. First, three experimental animals of each of the three species were selected in good health. Subsequently, the experimental animals were dissected and several indices of the designated intervertebral discs and the height of the adjacent vertebrae were measured. At the same time, the ease of the surgical operation was assessed intraoperatively (in terms of the ease of preoperative localization, the clarity of the intraoperative structures, and the length of the surgical operation). In addition, the animal type most suitable for the construction of a spinal fusion surgery model was evaluated. Finally, a rabbit spinal fusion surgical model was constructed and evaluated by palpation and gross view of the tissue to assess the degree of spinal fusion. The data on the disc structure of rabbits have more advantages than those of mice and rats in constructing surgical models, with easier preoperative localization, more precise intraoperative structure, and shorter surgery time. Spinal fusion surgery in rabbits was successful, with a significant trend toward fusion at 4 weeks postoperatively. Rabbits were superior to mice and rats as experimental animals for establishing spinal fusion surgery models, and the fusion models were effective.

## Graphical abstract

In this study, spinal fusion models in mice, rats and rabbits are compared and discussed. The results show that after comparing the anatomical structure and the complexity of surgical operation, among the three experimental animals in this study, the rabbit as an experimental animal is superior to the mouse and the rat in the establishment of spinal fusion surgical model. In addition, the feasibility of using New Zealand white rabbits to establish a spinal fusion model was high, and the operation is clear and simple, and the modeling effect is good.

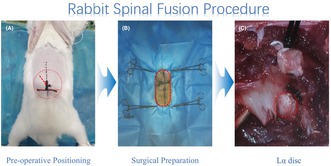

## 1 INTRODUCTION

In recent years, novel graft materials and interbody fusion devices have shown great potential in promoting spinal fusion, but they must undergo safety and efficacy evaluation in multiple animal models before they can be used in the clinic. Therefore, it is particularly important to select suitable experimental animals to construct spinal fusion surgery models, which is a necessary step toward achieving clinical translation. At present, a variety of mammals have been successfully used to construct surgical models of spinal fusion. Qin et al.^1^ successfully constructed a spinal fusion surgical model in the cervical spine of goats and confirmed that goats provide an excellent animal model for biomechanical studies of the cervical spine. In addition, several studies have shown that the cervical vertebrae of sheep and goats are similar in structure to the human spine, which also makes them a suitable choice for the construction of spinal fusion surgical models.^2^, ^3^, ^4^ A number of studies have meanwhile successfully constructed spinal fusion surgical models in the monkey spine, with better modeling results than in goats and sheep.^5^, ^6^, ^7^ Dogs and pigs have also been used to establish animal models of spinal fusion^8^; however, complications including cage subsidence, implant failure, and adjacent segment degeneration occur at higher rates in canine spinal fusion experiments,^9^, ^10^ and pigs have a similar problem.^11^ In addition, numerous studies have been devoted to improving the above mammalian spinal fusion models, with some success.^8^, ^12^ Although the biomechanical properties of the cervical spines of these mammals are similar to those of the human cervical spine (Table 1),^8^ they are not suitable for a large number of experimental studies because they are expensive or protected animals, or the surgical conditions are too demanding.^8^ Given the limitations of research using large animals, small animal spinal fusion models have become the preferred choice for spinal research.

**TABLE 1:** Vertebral morphometry measurement reported in different studies.

Parameter	Human	Pig	Baboon	Sheep	Goat
IVD height (mm)	11.07 ± 2.31	5.12 ± 0.96	5.32 ± 2.44	3.80 ± 0.42	4.09 ± 0.68
Vertebral body width (mm)	49.01 ± 4.62	37.57 ± 2.85	36.23 ± 6.04	27.03 ± 2.19	24.30 ± 2.02
Vertebral body depth (mm)	36.36 ± 3.34	25.28 ± 2.04	22.69 ± 1.46	19.18 ± 1.43	18 ± 1.65

In small animal spinal fusion models, mice, rats, and rabbits are the three most commonly used experimental animals. Compared to other experimental animals, they offer advantages such as ease of surgical manipulation, low cost, and ease of breeding. There have also been studies using these three animals for surgical modeling,^13^, ^14^ but they have not been evaluated and compared. In order to remedy this shortcoming and at the same time provide a wider choice of animal models, our study compares the anatomy of three commonly used experimental animals from the point of view of experimental animal selection for spinal fusion surgical models. We comprehensively compared the anatomical data of the three animal species. Subsequently, based on the data analysis, we selected rabbits whose intervertebral disc length, width, and height ratios most closely resemble those of human discs. We then surgically established a standardized rabbit spinal fusion model and finally evaluated its feasibility as a spinal fusion surgical model.

## 2 MATERIALS AND METHODS

### 2.1 Experimental animals

The experimental animals selected for initial comparison were: 3 SPF mice (age 3 months), 3 rats (age 3 months) and 3 New Zealand white rabbits (age 3 months). For the establishment of a rabbit spinal fusion surgery model, 6 New Zealand white rabbits (age 3 months) were used.

### 2.2 Main reagents and instruments

The following reagents were used: 3% pentobarbital sodium (Sinopharm, China), 2% lidocaine (Zhuofeng Pharmaceutical, China), 0.9% sodium chloride solution (Fuzheng Pharmaceuticals, China), penicillin potassium for injection (Yuxin Biologicals, China), and 4% paraformaldehyde (Huapu Biologicals, China). Surgical instruments included: a bone graft fusion device (Yoyuan Medical, China), vernier calipers (Xigong Measurement, China), aseptic syringes (Zhenkang Medical, China), and an electronic precision balance (Mettler Toledo, Switzerland).

### 2.3 Experimental groupings

Group A comprised 3 mice, Group B 3 rats, Group C 3 New Zealand white rabbits, and Group D 6 New Zealand white rabbits (numbered D1, D2, D3, D4, D5 and D6). We observed all experimental animals selected for this experiment for 5–7 days. The main observation indexes included whether the experimental animals were well developed, whether there were any deformity, whether there was thickness of the lumbar back, whether there was protrusion of the vertebrae in the lumbar back, and whether there was any independent activity, movement, or response to external stimuli, and gait. The animals in Groups A, B and C were weighed and anesthetized after observing that there was no abnormality in the above indexes. For euthanasia, the animals in Groups A, B and C were injected with an excessive amount of 3% sodium pentobarbital solution (mice and rats: intraperitoneal injection, ≥ 3 × 1.0–1.5 mL/kg; rabbits: intravenously at the margin of the ear, ≥ 3 × 1.0 mL/kg). Animals in Group D were anesthetized by injection of a regular amount of 3% sodium pentobarbital solution (intravenously at the margin of the ear, ≥ 1.0 mL/kg). The experimental animals are shown in Table 2.

**TABLE 2:** Grouping and treatment of experimental animals.

Group	Animal name	Number	Handling
A	SPF Mice	3	Euthanasia after 1 week of observation
B	SPF Rats	3	Euthanasia after 1 week of observation
C	New Zealand White Rabbit	3	Euthanasia after 1 week of observation
D	New Zealand White Rabbit	6	Surgical modeling of rabbit spinal fusion, evaluation followed by execution

#### 2.3.1 Physiological structure measurement

**FIGURE 1:**
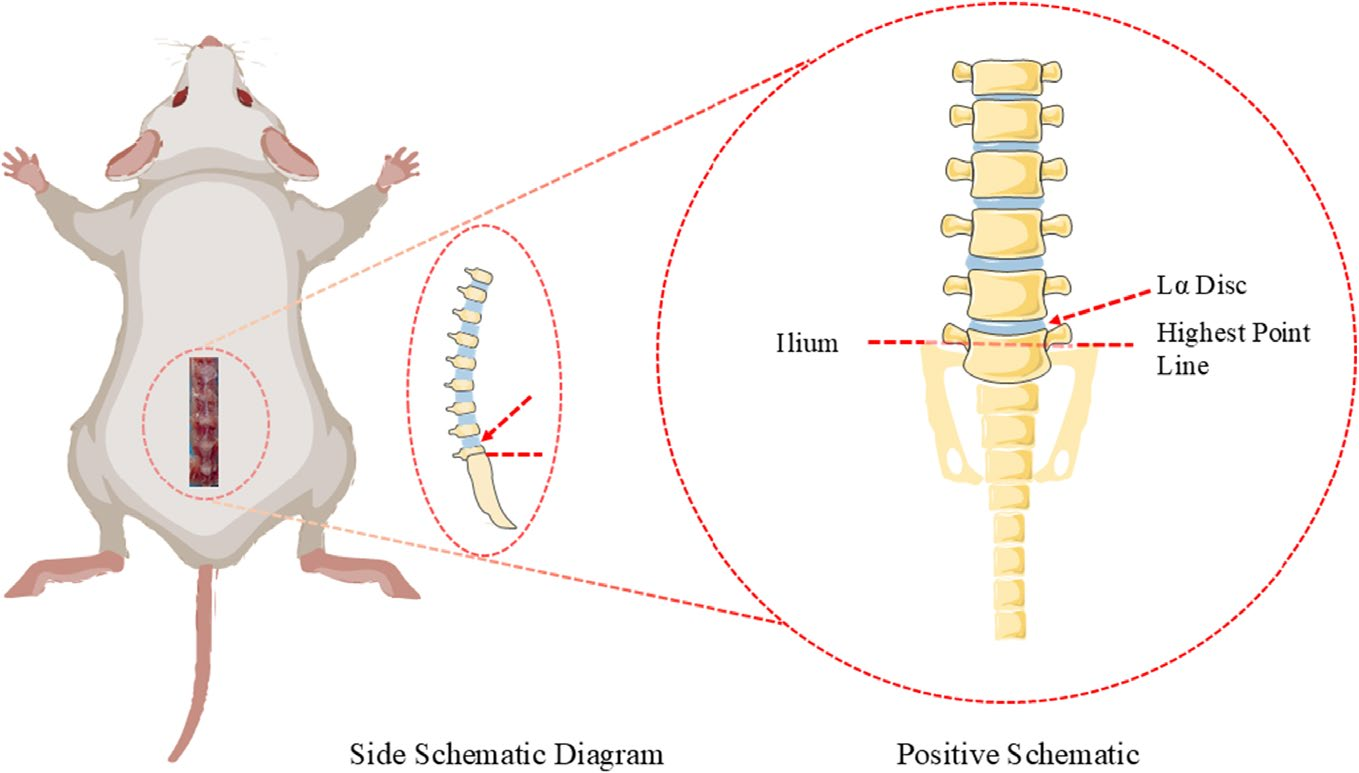
The Lα intervertebral disc is the disc above the midpoint of the line connecting the highest points of the iliac bones.

After the experimental animals in Groups A, B and C were killed, the limbs were fixed to the surgical table, and the midpoint of the line connecting the highest points of the iliac bones in the back of both sides was taken as the center and marked with a locating needle. A longitudinal skin incision (3 cm in mice, 4 cm in rats, and 5 cm in rabbits) perpendicular to the connecting line was made through the marked point, and the intervertebral discs were separated and exposed one by one until the first disc above the point marked by the locating needle was exposed completely and labeled as the Lα intervertebral disc. The Lα intervertebral disc and adjacent upper and lower vertebrae were removed, quickly rinsed with 0.9% NaCl solution and dried, and the height of the Lα intervertebral disc, the length of the transverse/longitudinal diameter of the Lα intervertebral disc and the heights of the adjacent upper and lower vertebral bodies were measured directly with vernier calipers (0.05 mm) and recorded; the volume of the nucleus pulposus was also measured indirectly with a 1‐mL syringe containing 1 mL of 0.9% NaCl solution and recorded. The localization of the Lα intervertebral disc is shown in Figure 1.

#### 2.3.2 Rabbit spinal fusion surgery model

**FIGURE 2:**
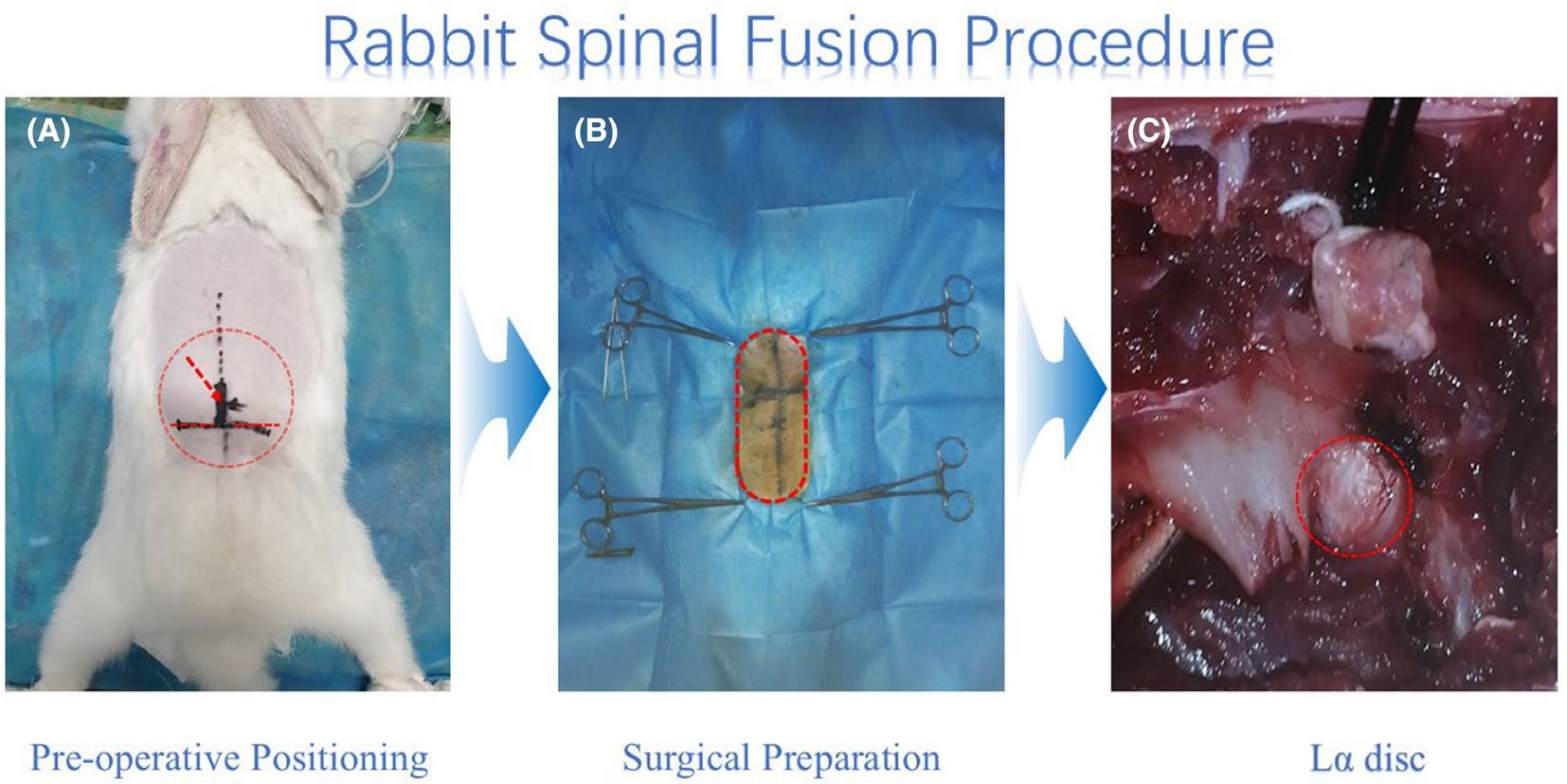
Establishment of rabbit spinal fusion surgery model. Panel A shows the preoperative localization marking after skin preparation; panel B shows the preparation before the start of the surgery and panel C shows the intraoperative isolation of the Lα intervertebral disc.

Group D New Zealand White rabbits were placed on the animal anesthesia table and then injected intravenously with 3% pentobarbital sodium solution (1.0 mL/kg) through one ear margin to perform general anesthesia. After complete anesthesia, the limbs of the rabbits were fixed on the surgical table, and the midpoint of the line connecting the highest points of the iliac bones in the dorsal region of the bilateral backs was taken as the center point and marked with a locating needle. The skin was prepared with a shaving device, and the surgical area was disinfected with iodine‐vapor disinfectant using a sterile cavity towel (Figure 2A and 2B). A longitudinal skin incision (5 cm) was made perpendicular to the connecting line, and the dorsal dermatomes, dorsal lumbar longest muscles, and muscles of the right side of the spine were separated downward, fully exposing the Lα intervertebral disc on the right side of the back, located above the line connecting the highest points of the iliac bone (the first intervertebral disc above the line is referred to as the Lα intervertebral disc) (Figure 2C). At the same time, the vertebral spinous processes were removed and any adherent soft tissues were completely separated The spinous processes were then ground into small particles with a bone grinder. The fibrous annulus of the Lα intervertebral disc was disrupted with a 10 mL sterile syringe needle (0.2–0.3 cm in length), and the nucleus pulposus and adjacent cartilaginous endplates within the Lα intervertebral disc were removed with a scraping spoon. Based on the measured volume of the nucleus pulposus, a sufficient amount of ground sphenoidal bone particles was inserted into the space created by removing the nucleus pulposus using a bone powder conveyor, and the annulus fibrosus of the Lα intervertebral disc defect was sealed with bone wax. The surgical incision was closed layer by layer after surgery. The surgical procedure is shown in Figure 2.

#### 2.3.3 Postoperative treatment and evaluation

After surgery, the rabbits were housed individually in separate cages, fed and watered freely, and injected with sodium penicillin and buprenorphine for 3 consecutive days for postoperative anti‐inflammatory and analgesic purposes. The rabbit spinal fusion surgical model was evaluated by manual palpation at 1 week postoperatively and 3 weeks postoperatively.^15^, ^16^ The stitches were removed at the appropriate time based on the healing of the surgical incision. At 4 weeks postoperatively,^17^ the animals were euthanised with an anesthesia overdose, and the spinal segments containing the Lα intervertebral discs were taken to observe the tissue compatibility.

Six orthopedic surgeons not involved in this experiment were randomly selected to perform manual palpation and assessment of the spinal fusion segments in rabbits at 1 week and 3 weeks post‐surgery. Evaluation criteria were as follows: spinal fusion was recorded as present (+) if ≥ 4 surgeons confirmed it, while spinal fusion was recorded as absent (−) if < 4 surgeons confirmed it.

## 3 RESULTS

### 3.1 Measurement of physiological structure data of three experimental animals

**FIGURE 3:**
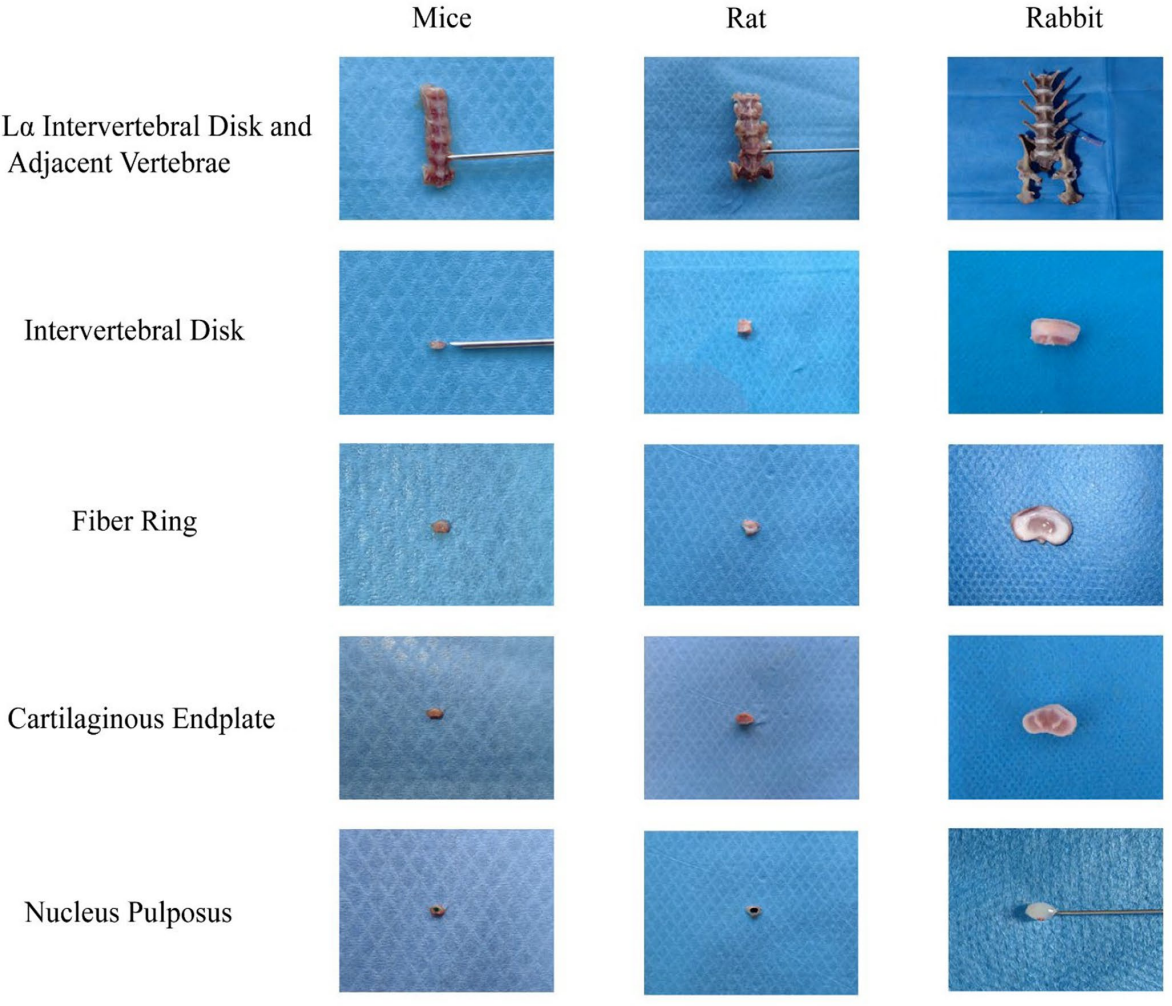
Photographs of the relevant structures of the intervertebral discs of mice, rats and rabbits. Vertically, from top to bottom, the panels show the Lα intervertebral disc and its adjacent vertebral bodies, intervertebral discs, annulus fibrosus, cartilaginous endplates, and nucleus pulposus for each type of animal, while horizontal panels show a comparison of each structure in (left to right) mice, rats, and rabbits.

The results of the process of dissecting the Lα intervertebral disc and adjacent vertebrae in the three animals show that preoperative intervertebral disc localization in rats and New Zealand white rabbits is simpler and more accurate than preoperative intervertebral disc localization in mice. The surgical dissection was easiest in New Zealand rabbits, more difficult in rats and most difficult in mice. The anatomy of the Lα intervertebral disc and adjacent vertebrae in the three animals is shown in Figure 3. The overall size of the Lα intervertebral disc and adjacent vertebrae varied based on the size of the animals. More specifically, the mice Lα intervertebral discs are significantly smaller than the Lα intervertebral discs of rats and New Zealand rabbits, with the Lα intervertebral disc of the rabbit being the largest. The annulus fibrosus is arranged in concentric circles in all three animals, and the structure of the annulus fibrosus is smallest in mice, larger in rats, and largest New Zealand rabbits. Further, the cartilaginous endplates are smallest and most difficult to separate in mice. The cartilage endplates of rats are larger than those of mice but significantly smaller than those of New Zealand rabbits. The cartilaginous endplates of the rabbit are the easiest to separate. The nuclei of all three animals are milky‐white, transparent and gelatinous. The amount of medulla in New Zealand rabbits is greater than that in rats and mice. The mice have the lowest amount of nucleus pulposus. Measurements of the Lα intervertebral discs and adjacent vertebrae of the three animals are shown in Table 3.

**TABLE 3:** Measurements of Lα intervertebral disc and adjacent vertebral body heights in mice, rats and New Zealand rabbits (means ± SD).

Animals	Weight (kg)	Lα disc height (mm)	Lα disc transverse diameter (mm)	Lα disc longitudinal diameter (mm)	Upper vertebral height (mm)	Lower vertebral height (mm)	Nucleus volume (mL)
Mice	0.032	0.90 ± 0.05	1.73 ± 0.08	0.71 ± 0.08	3.80 ± 0.25	2.90 ± 0.33	0.023 ± 0.008
Rat	0.313	2.27 ± 0.23	4.67 ± 0.13	2.63 ± 0.13	6.26 ± 0.28	6.99 ± 0.57	0.057 ± 0.004
Rabbit	1.844	7.86 ± 0.37	15.02 ± 0.40	8.13 ± 0.40	14.33 ± 0.73	12.32 ± 0.74	0.088 ± 0.008

### 3.2 Evaluation of the rabbit spinal fusion surgical model

**FIGURE 4:**
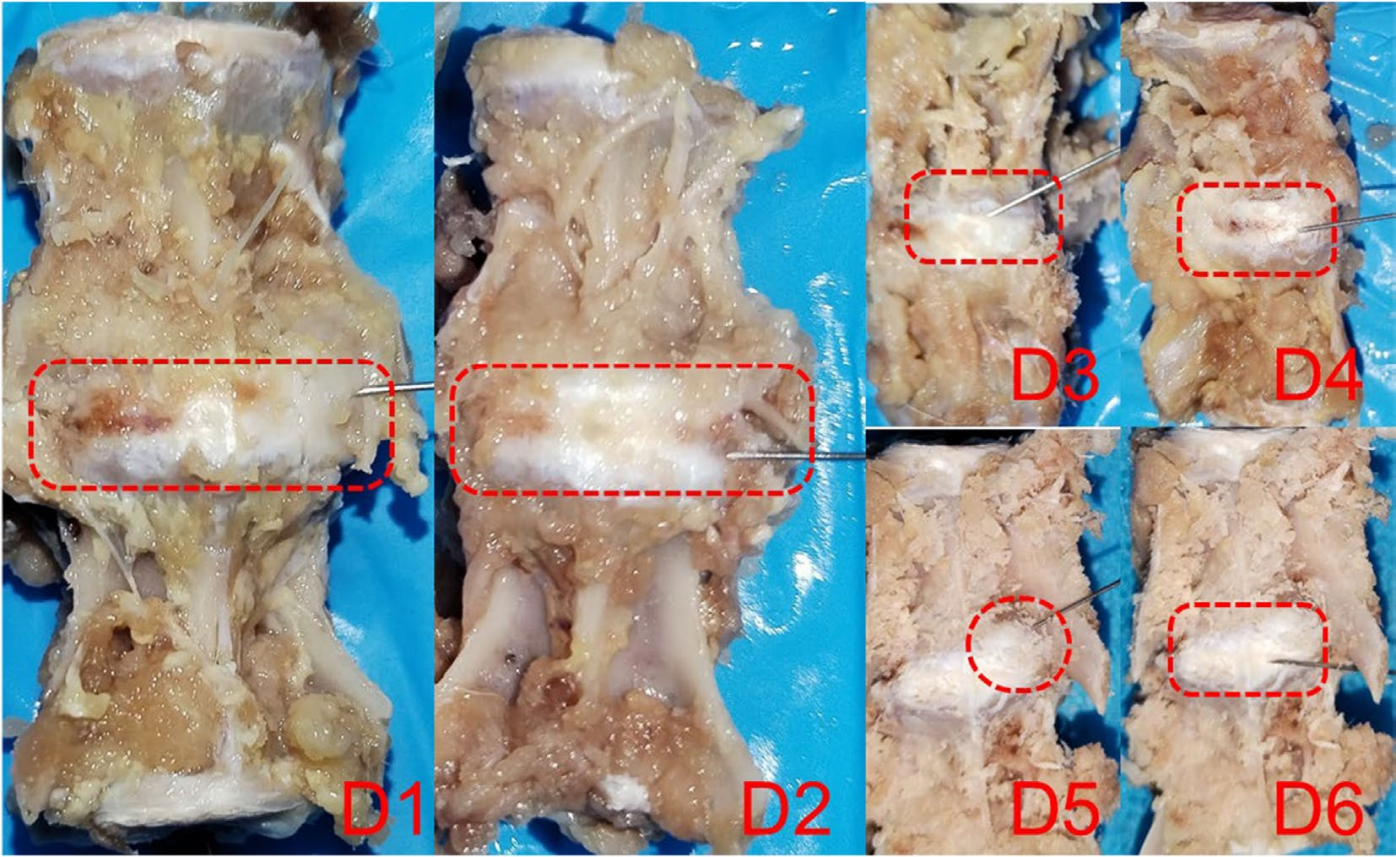
4‐week postoperative visualization of rabbit spinal fusion: D1, D2, D3, D4, and D6 all show a trend toward bony fusion, and D5 shows a prominent growth of a partial bone graft.

After comprehensively comparing the advantages and disadvantages of the Lα intervertebral disc and adjacent vertebrae of the three animals used in this experiment, New Zealand rabbits were selected as the research subjects to establish the spinal fusion surgical model. The stability of the spinal fusion segments and the fusion status of the rabbits at 1 week and 3 weeks postoperatively were evaluated by manual palpation, respectively. The results show that no fusion was palpated at 1 week and 3 weeks postoperatively, but the spinal fusion segments are stable. The Lα disc fusion at 4 weeks postoperatively is shown in Figure 4.

## 4 DISCUSSION

Anatomical measurements and evaluations confirmed that all three of these experimental animals can be used for spinal fusion surgical modeling, but with varying degrees of difficulty and impact on the experiment. Several previous studies have also confirmed this idea.^17^, ^18^, ^19^, ^20^ Kang et al.^13^ successfully constructed a spinal fusion model in the rat spine, but it had significant postoperative complications and the spine was prone to fusion on its own after surgery, which seriously affected the experimental results. The lumbar spine fusion model in mice has also been successfully constructed and explored, but because of the greater difficulty of the surgical operation and a complicated fusion process mice are not the first choice for a spinal fusion surgical model.^21^, ^22^ Virk et al.^17^ have outlined the advantages and disadvantages of the various animal models used to study spinal fusion and have also described the New Zealand White Rabbit, which is the most popular preclinical model for studying spinal fusion. Further, they also outlined the key steps required to plan and perform spinal fusion surgery in this model. In addition, Cui et al.^23^ successfully demonstrated a positive role of bone marrow mesenchymal stem cells (BMSCs) in spinal interbody fusion by establishing a rabbit cervical vertebral body fusion model. All these results indicate that rabbits are superior to mice and rats as a spinal fusion surgical model and are effective experimental subjects for constructing spinal fusion surgical models.

In this study, mice, rats, and New Zealand white rabbits were selected for the establishment of an animal model of spinal fusion. We compared the anatomical processes of the corresponding Lα intervertebral discs and adjacent vertebrae in mice, rats, and New Zealand white rabbits and the Lα intervertebral disc and adjacent vertebral structures of the three animals. Our data indicate that New Zealand rabbits appear to be more suitable for establishing spinal fusion surgery models compared to rats and mice. Specifically, the spine, vertebrae, and intervertebral discs of mice and rats are small, and the intervertebral space after removal of the intervertebral discs is very narrow, and the upper and lower vertebrae are prone to spontaneous fusion.^17^, ^24^ During dissection to obtain the Lα disc and adjacent vertebrae, preoperative Lα disc localization is more difficult and less accurate in mice than in rats and New Zealand white rabbits, and it takes longer to dissect the target structures in mice than rats, mainly due to the small size and light weight of mice and the smaller iliac bones on both sides. In addition, the experimental participants' lack of clarity about the anatomical structures in mice may be another important factor. The Lα intervertebral disc and adjacent vertebral bodies in rats are larger than the vertebral bodies and Lα intervertebral structures in mice, and surgical intervention to disrupt the disc structure is feasible. However, after removal of the Lα intervertebral disc in rats, the intervertebral space is narrower than in rabbits, making surgical manipulation for bone grafting more difficult, which is consistent with the findings of Virk et al.^17^ Therefore, the rat is not the best spinal fusion modeling object for this experiment.

The rabbit spinal fusion surgery model established in this exploratory study demonstrated favorable outcomes under subjective observation. The reason for the failure to reach fusion on manipulative palpation at 1 week and 3 weeks postoperatively is most likely due to insufficient time. The histomicrographs of the model rabbits at 4 weeks postoperatively show a clear trend toward fusion, explaining why fusion was not palpated when insufficient time was allowed after previous manipulative palpation. This indicates that the spinal fusion surgical procedure performed on rabbits in this study is highly effective, and the established rabbit spinal fusion model appears to be a suitable animal model for spinal experimental research. However, our study does not employ objective measurement methods such as radiographic or histological analysis, relying instead on subjective assessments based on manual evaluation. Although we ensured that the evaluators strove for fairness and objectivity in their assessments, the absence of objective measurement methods means our conclusions are not optimal experimental findings. Therefore, our conclusions carry certain limitations and are applicable only for comparative studies in small experimental animal models of spinal fusion surgery.

In addition, the rabbit iliac bone is much larger than that of rats and mice, and can provide sufficient bone graft for intervertebral fusion. The New Zealand Greater White rabbit has a larger intervertebral space and vertebral body compared with mice and rats, which makes it easier to operate surgically, more convenient for internal fixation, and better simulates the biomechanics of the human spine. Therefore, our study shows that it is the most suitable choice for constructing a spinal fusion surgery model.

Overall, preoperative disc localization is easier and more accurate in rabbits than in mice and rats, and the intraoperative structure of rabbits is clear, which reduces the difficulty of surgical operation. In addition, the cartilage endplates of rabbits are easier to separate and the bone grafting operation is simpler, all of which favors the establishment of spinal fusion model. After comprehensively comparing anatomical structures and the complexity of surgical procedures, our data indicate that among the three experimental animal subjects in this study rabbits are the superior experimental animal for establishing spinal fusion surgery models. In addition, the feasibility of using New Zealand white rabbits to establish a spinal fusion model is high, the operation is clear and simple, and the modeling effect is good. Nevertheless, our study comparing data from the three experimental animal models in the establishment of spinal fusion models has certain limitations, including a small sample size, lack of biomechanical testing, and a short follow‐up period.

## AUTHOR CONTRIBUTIONS

**Jiang hong Lu:** Writing – review and editing. **Yi cun Hu:** Investigation; methodology. **Xin Yao:** Data curation; formal analysis; funding acquisition; writing – original draft.

## FUNDING INFORMATION

This work is not backed by funds.

## CONFLICT OF INTEREST STATEMENT

The authors have no relevant affiliations or financial involvement with any organization or entity with a financial interest in or financial conflict with the subject matter or materials discussed in the manuscript. This includes employment, consultancies, honoraria, stock ownership or options, expert testimony, grants or patents received or pending, or royalties. No writing assistance was utilized in the production of this manuscript.

## ETHICS STATEMENT

The above experimental animals are obtained from Lanzhou Institute of Veterinary Medicine, Chinese Academy of Agricultural Sciences, with laboratory animal license [SCXK (Gan) 2020‐0002]. This study is approved by the Animal Ethics Committee of the Second Hospital of Lanzhou University (Approval No. D2023‐144).

## Data Availability

The datasets used or analyzed during the current study are available from the corresponding author upon reasonable request.
